# A case of anterior choroidal artery occlusion test under MEP monitoring for a recurrent internal carotid artery–anterior choroidal artery bifurcation aneurysm clipping

**DOI:** 10.1093/jscr/rjac639

**Published:** 2023-01-26

**Authors:** Yosuke Suzuki, Kosumo Noda, Souichirou Yasuda, Yasuaki Okada, Syun Ono, Katsunari Kiko, Kohei Yoshikawa, Norio Miyoshi, Tomomasa Kondo, Kenichi Haraguchi, Jyunpei Oda, Nakao Ota, Hiroyasu Kamiyama, Sadahisa Tokuda, Rokuya Tanikawa

**Affiliations:** Stroke Center, Department of Neurosurgery, Sapporo Teishinkai Hospital, Sapporo, Hokkaido, Japan; Stroke Center, Department of Neurosurgery, Sapporo Teishinkai Hospital, Sapporo, Hokkaido, Japan; Stroke Center, Department of Neurosurgery, Sapporo Teishinkai Hospital, Sapporo, Hokkaido, Japan; Stroke Center, Department of Neurosurgery, Sapporo Teishinkai Hospital, Sapporo, Hokkaido, Japan; Stroke Center, Department of Neurosurgery, Sapporo Teishinkai Hospital, Sapporo, Hokkaido, Japan; Stroke Center, Department of Neurosurgery, Sapporo Teishinkai Hospital, Sapporo, Hokkaido, Japan; Stroke Center, Department of Neurosurgery, Sapporo Teishinkai Hospital, Sapporo, Hokkaido, Japan; Stroke Center, Department of Neurosurgery, Sapporo Teishinkai Hospital, Sapporo, Hokkaido, Japan; Stroke Center, Department of Neurosurgery, Sapporo Teishinkai Hospital, Sapporo, Hokkaido, Japan; Stroke Center, Department of Neurosurgery, Sapporo Teishinkai Hospital, Sapporo, Hokkaido, Japan; Stroke Center, Department of Neurosurgery, Sapporo Teishinkai Hospital, Sapporo, Hokkaido, Japan; Stroke Center, Department of Neurosurgery, Sapporo Teishinkai Hospital, Sapporo, Hokkaido, Japan; Stroke Center, Department of Neurosurgery, Sapporo Teishinkai Hospital, Sapporo, Hokkaido, Japan; Stroke Center, Department of Neurosurgery, Sapporo Teishinkai Hospital, Sapporo, Hokkaido, Japan; Stroke Center, Department of Neurosurgery, Sapporo Teishinkai Hospital, Sapporo, Hokkaido, Japan

**Keywords:** anterior choroidal artery, internal carotid artery, surgical clipping, intracerebral aneurysm, motor-evoked potential monitoring, occlusion test

## Abstract

A 59-year-old female with recurrent Anterior Choroidal Artery (AchA) aneurysm was elected for surgery at our institution through a standard pterional approach. Two thin perforating branches were found to origin from the dome of the aneurysm during operation, and therefore complete aneurysm clipping preserving these branches was not feasible. These perforating branches were temporarily occluded under motor-evoked potential (MEP) monitoring. The MEPs remained stable during 10 min of temporary clipping, and we concluded that these branches could be sacrificed, and therefore neck clipping was performed occluding these tiny AchA perforators. Although postoperative magnetic resonance imaging with diffusion-weighted images showed ischemic signs in left AchA territory after the operation, the patient remained asymptomatic and was discharged home with mRS 0.

## INTRODUCTION

Anterior choroidal artery (AchA) occlusion can result in contralateral hemiplegia, contralateral hemi-hypoesthesia and homonymous hemianopsia. Despite technological advance in endovascular and surgical techniques, AchA aneurysms remain challenging especially for its difficulty to preserve all of the AchA perforators. As occlusion of any of these small branches can lead to poor outcomes, a complete neck clipping is usually impossible when they originate from the dome of the aneurysm.

The authors report a case of a left recurrent internal carotid artery (ICA)–AchA bifurcation aneurysm. A complete neck clipping was performed, occluding small perforating branches originating from the dome with an aid of motor-evoked potential (MEP) monitoring.

## CASE REPORT

**Figure 1:**
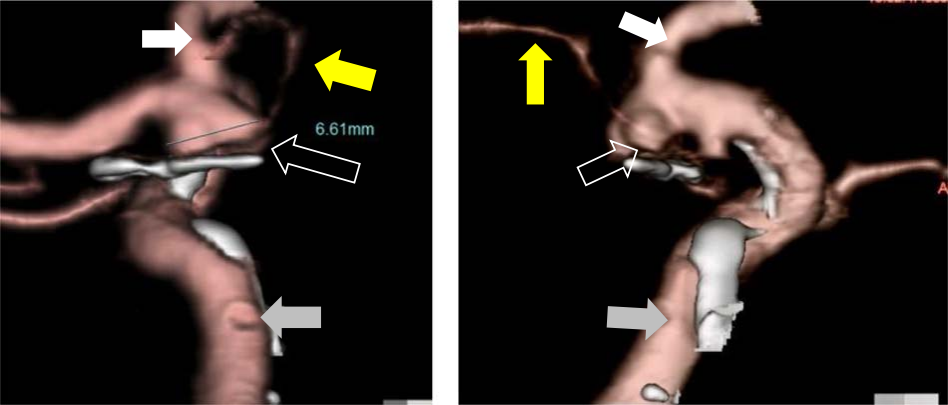
Preoperative CT angiography showed recurrent left ICA–AchA aneurysm and a surgical clip of the previous operation (open white arrow). The yellow arrow points to the AchA main trunk originating from ICA. The white and gray arrows indicate the anterior cerebral artery and ICA, respectively.

A 59-year-old woman was admitted to our hospital with a history of surgical clipping of an unruptured AchA aneurysm 9 years ago. The aneurysm was incompletely obliterated because of perforating branches arising from the aneurysm dome. A computer tomography (CT) angiogram revealed regrowth of the aneurysm to size with a maximum dome diameter of 6.6 mm (Fig. 1). Neurological examination at admission showed no neurological deficit.

Despite the risk of postoperative neurological deficit, the patient chose surgical clipping of the recurring aneurysm as her treatment.

**Figure 2:**
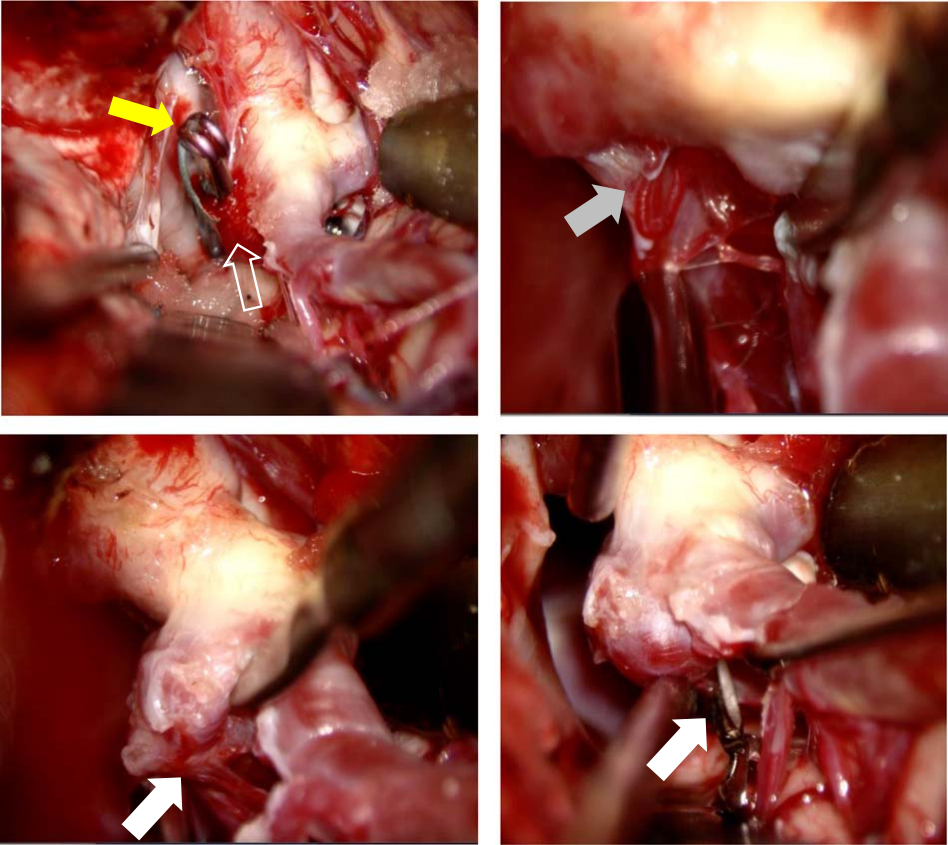
After Sylvian fissure dissection, the recurrent aneurysm (open white arrow) and the surgical clip (yellow arrow) were exposed. AchA’s main trunk (gray arrow) was detached from the aneurysm (compressed by microscissors), and the AchA main trunk was confirmed to arise from the ICA. Two small AchA branches were also found arising from the aneurysmal dome (white arrow). The two small AchA branches were temporarily occluded for 10 min.

The patient was put under general anesthesia and MEP recordings were performed with needle electrodes for transcranial electrical stimulation placed on the scalp at the C3 and C4, according to the International 10-20 System. Stimulation (rate: 0.5 Hz, intensity: 70–90 mA, duration: 0.2 ms) was performed with train of six pulses with inter stimulus intervals of 2 ms. Compound muscle action potential was recorded from the bilateral abductor pollicis brevis and contralateral abductor hallucis, using a pair of needle electrodes. The patient was placed supine with the head rotated 15° to the right side fixed with a three-pin head frame. The surgery started with cervical dissection to secure the left common carotid artery and ICA for proximal control. The external carotid artery and superior thyroid artery were also dissected to enable retrograde suction decompression (RSD). Then, a curved skin incision was made following the previous surgical scar, and a frontotemporal craniotomy was performed. A distal Sylvian fissure dissection approach was performed to expose the carotid bifurcation, recurrent ICA–AchA aneurysm and the surgical clip. AchA’s main trunk was detached from the aneurysm dome while performing RSD, but two thin perforating branches were found arising from the aneurysmal dome (Fig. 2).

**Figure 3:**
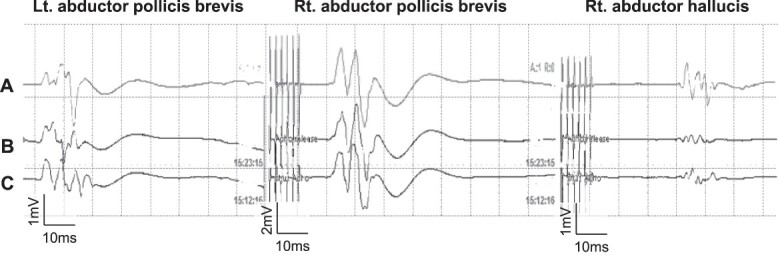
The MEP did not change even after 10 min of occlusion and until completion of skin closure. (**A**; after skin is closed, **B**: after 10 min of occlusion, **C**; before occlusion of Ach A branches).

Although a complete aneurysm clipping preserving these branches was not feasible, it is also known from a report by Ihm *et al*. [1] that an incomplete neck clipping of a recurring aneurysm has a higher risk of recurrence. The authors performed temporary occlusion of these small branches under MEP monitoring to determine whether it was possible to sacrifice any of these perforators arising from the aneurysmal dome. MEP recording was performed every 1 min with no MEP amplitude change up to 10 min after temporally clipping of the perforators (Fig. 3).

**Figure 4:**
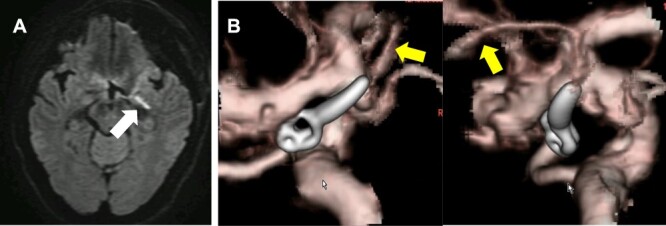
DWI-MRI revealed a new ischemic brain lesion at the uncus of the left AchA territory 1 day after surgical clipping (**A**). CTA demonstrated complete aneurysm obliteration and a healthy patency of the AchA main trunk (**B**, yellow arrow).

The authors were able to determine that the perforating branches originating from the dome could be sacrificed and therefore allowing complete neck clipping. And MEP did not change until skin was closed (Fig. 3). Although postoperative diffusion-weighted images (DWI) on magnetic resonance imaging (MRI) showed hyperintensity signals at the medial side of the uncus (Fig. 4A), the patient remained asymptomatic and was discharged home with a modified Rankin Scale of 0. Postoperative CT angiogram showed complete aneurysm obliteration and patency of the AchA main trunk (Fig. 4B).

## DISCUSSION

Surgical clipping of ICA–Ach aneurysms has a higher complication rate than clipping of other aneurysms [2–5], especially for those aneurysms with a perforating branch arising from the dome as reported by Friedman *et al*. [6]. Usually, guaranteeing a good clinical outcome is more important than pursuing complete neck clipping, especially for unruptured intracranial aneurysms [5, 7]. However, complete aneurysm occlusion should be pursued for recurrent aneurysms to reduce the risk of another recurrence requiring repetitive treatment. Thus, when treating a recurrent aneurysm with a high risk of neurological morbidity, as in our case, it is of paramount importance to foresee the consequences of each surgical manipulation.

Suzuki *et al*. [8] reported that MEP monitoring is safe and reliable for detecting AchA’s intraoperative blood flow insufficiency. Tanabe *et al*. [9] reported that the MEP decreased within 3.5 min after an AchA involving ICA aneurysm was temporarily occluded. Kameda *et al*. [10] also reported that maximal safe temporary clipping time was 5 min. Thus, no decrease in MEP amplitude after 10 min of occlusion of AchA would indicate that AchA is not likely supplying the internal capsule territory. However, the authors strongly recommend continuing MEP monitoring until the skin is closed and the surgery is finished, as there is no evidence in the literature describing a reliable temporal threshold for perforating branches test occlusion.

## CONCLUSION

The MEP-monitored AchA occlusion test is useful in determining whether AchA originating from the dome can be occluded.

## Data Availability

Data sharing is not applicable to this article as no datasets were generated or analyzed during the current study.
